# LRP1 Functions as an Atheroprotective Integrator of TGFβ and PDGF Signals in the Vascular Wall: Implications for Marfan Syndrome

**DOI:** 10.1371/journal.pone.0000448

**Published:** 2007-05-16

**Authors:** Philippe Boucher, Wei-Ping Li, Rachel L. Matz, Yoshiharu Takayama, Johan Auwerx, Richard G.W. Anderson, Joachim Herz

**Affiliations:** 1 Institut Gilbert-Laustriat, UMR 7175 LC-1, Department of Pharmacology; Centre National de la Recherche Scientifique/Institut National de la Santé et de la Recherche Medicale/Université Louis Pasteur, Illkirch, France; 2 Department of Cell Biology, University of Texas Southwestern Medical Center, Dallas, Texas, United States of America; 3 Institut de Génétique et de Biologie Moléculaire et Cellulaire, Centre National de la Recherche Scientifique/Institut National de la Santé et de la Recherche Medicale/Université Louis Pasteur, Illkirch, France; 4 Department of Molecular Genetics, University of Texas Southwestern Medical Center, Dallas, Texas, United States of America; RIKEN Genomic Sciences Center, Japan

## Abstract

**Background:**

The multifunctional receptor LRP1 controls expression, activity and trafficking of the PDGF receptor-β in vascular smooth muscle cells (VSMC). LRP1 is also a receptor for TGFβ1 and is required for TGFβ mediated inhibition of cell proliferation.

**Methods and Principal Findings:**

We show that loss of LRP1 in VSMC (smLRP^−^) in vivo results in a Marfan-like syndrome with nuclear accumulation of phosphorylated Smad2/3, disruption of elastic layers, tortuous aorta, and increased expression of the TGFβ target genes thrombospondin-1 (TSP1) and PDGFRβ in the vascular wall. Treatment of smLRP1^−^ animals with the PPARγ agonist rosiglitazone abolished nuclear pSmad accumulation, reversed the Marfan-like phenotype, and markedly reduced smooth muscle proliferation, fibrosis and atherosclerosis independent of plasma cholesterol levels.

**Conclusions and Significance:**

Our findings are consistent with an activation of TGFβ signals in the LRP1-deficient vascular wall. LRP1 may function as an integrator of proliferative and anti-proliferative signals that control physiological mechanisms common to the pathogenesis of Marfan syndrome and atherosclerosis, and this is essential for maintaining vascular wall integrity.

## Introduction

Cholesterol-induced atherosclerosis is a major cause of morbidity and mortality in Western societies. In the liver, the low-density lipoprotein (LDL) receptor functions in concert with another LDL receptor family member, the LDL receptor-related protein 1 (LRP1), in removing cholesterol carrying lipoprotein particles from the bloodstream. However, in the smooth muscle cells of the artery wall LRP1 acts through a different, cholesterol-independent mechanism in the maintenance of vascular wall integrity and atherosclerosis prevention, which relies in part on the ability of LRP1 to regulate the activity and subcellular trafficking of the PDGFRβ [1]–[3]. SmLRP^−^ mice that lack LRP1 in vascular smooth muscle cells show thickening of the muscular layer and greatly increased susceptibility to atherosclerotic lesion development, even at low plasma cholesterol levels. On a high cholesterol diet, lesion development and progression is dramatically accelerated, resulting in the occlusion of major arteries within 2 months of cholesterol feeding. Pharmacologic inhibition of PDGFRβ signaling reduced both lesion progression and smooth muscle over-proliferation, suggesting that the abnormal activation of PDGFRβ is at least in part responsible for the accelerated lesion development and progression [1].

On the other hand, the characteristic disruption of elastic layers, elongation of the aortas and aneurysms are hallmarks of Marfan syndrome (MFS) [4], [5], an autosomal dominant genetic disease. MFS is caused by loss of function mutations in fibrillin-1, a microfibrillar protein that coats the surface of elastic fibers where it binds and immobilizes the TGFβ large latency complex (LLC) [6]. Haploinsufficiency of fibrillin-1 [7], [8] markedly changes the extracellular targeting, sequestration and turnover of latent TGFβ, resulting in pronounced activation of the growth factor and the signaling pathway [4], [5], [9]. A phenotypically similar Marfan-like syndrome is caused by loss of function mutations in TGFβ receptors 1 or 2 [10]–[12], which nevertheless result in a paradoxical activation of the pathway and nuclear accumulation of pSmad2 [10]. Thus, abnormal activation of TGFβ signaling appears to be a common feature of Marfan and Marfan-like disorders.

Recently, LRP1 was reported to be identical to TGFβR(V), which is coexpressed together with TGFβ receptors I, II and III. LRP1/TGFβR(V) is required for mediating the growth inhibitory response of TGFβ, in conjunction with Smad2/3 signaling through TGFβR(I) and (II) [13], [14]. TGFβ1 can induce the expression of connective tissue growth factor (CTGF), PDGF-B and PDGF receptors [15]–[19]. Activation of a PDGF/PDGFR autocrine loop by TGFβ mediates epithelial-mesenchymal transition and constitutes a crucial mechanism that enhances tumor metastasis [20] and the malignancy of gliomas [21]. All of these proteins are not only ligands for LRP1, they also promote smooth muscle cell proliferation [16], [22]. Furthermore, TSP1, another ligand for LRP1 and a TGFβ target gene [23], directly mediates the conversion of latent TGFβ to the active form [24], [25] and TSP1 binding to an LRP1/calreticulin complex regulates cell adhesion [26]. Thus, LRP1 is tightly involved in the control of growth regulating signaling processes that involve TGFβ, PDGF, and their receptors. This prompted us to investigate, whether abnormal activation of TGFβ signaling could be responsible for the striking disruption of the elastic fiber network, fibrosis, and accelerated atherosclerotic lesion development in the vascular wall of smLRP^−^ mice [1].

## Results

### Activation of TGFβ signaling in LRP1-deficient VSMC

**Figure 1:**
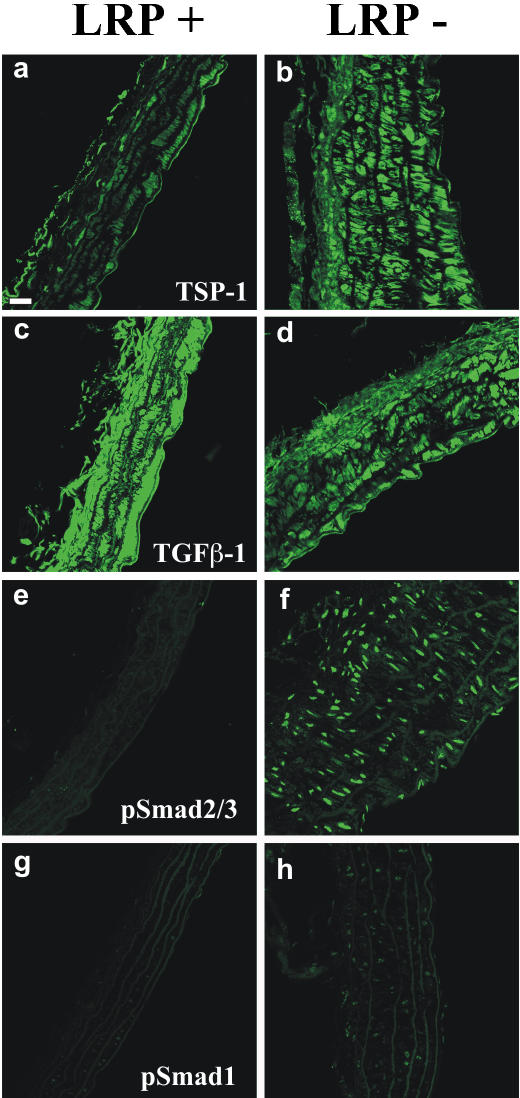
Increased pSmad2/3 expression and activation of TGFβ signaling in LRP^−^ mouse aorta. Longitudinal sections of abdominal aorta from SM22Cre^+^;LRP^flox/flox^;LDLR^−/−^ (LRP^−^) and LRP^flox/flox^;LDLR^−/−^ (LRP^+^) mice were stained with anti-TSP1, anti-TGFβ1, anti-pSmad2/3 and anti-pSmad1 antibodies. Reduced LRP1 expression results in greatly enhanced expression of pSmad2/3 and its target gene, TSP1. By contrast, TGFβ1 levels were slightly reduced, pSmad1 levels did not change. Bar in a indicates 20 µm.

Vascular wall fibrosis, disruption of elastic layers, and tortuousity of the aorta were suggestive of abnormal TGFβ signaling in smLRP-deficient mice. To obtain further evidence for the presence of increased TGFβ activity, we used immunohistochemistry to visualize the levels of TGFβ1 and TSP1, as well as the phosphorylated forms of Smad-1 and Smad-2, the intracellular mediators of TGFβ family signals, in aortas from cholesterol-fed, LDL receptor deficient mice expressing (LRP^+^) or not expressing LRP1 (LRP^−^) in their VSMC (Figure 1). Expression of TSP-1, a direct TGFβ1 target gene, was strikingly increased in LRP^−^ compared to LPR^+^ aortas (Panels a and b), whereas total TGFβ1 immunoreactivity was slightly reduced (Panels c and d), consistent with an increased activation and turnover of matrix-associated TGFβ.

TGFβ1 and other TGFβ family members, as well as angiotensin II are prominent inducers of Smad2-dependent target gene transcription, resulting in collagen deposition and disruption of the elastic layers in the vascular wall [27], [28]. Prominent accumulation of nuclear phosphorylated Smad2/3 (pSmad2/3) was found in VSMC in the absence of LRP1 (Panels e and f). This effect was specific for Smad2, which is the target of TGFβ signals, but not for Smad1, which responds to signals elicited by bone morphogenic proteins (BMPs). pSmad1 was not significantly elevated in the absence of LRP1 (Panels g and h). These findings, taken together with the molecular properties of LRP1, make it likely that TGFβ signaling in VSMC is indeed selectively increased in the absence of LRP1.

### Rosiglitazone reduces TGFβ signals in vitro and in vivo

**Figure 2:**
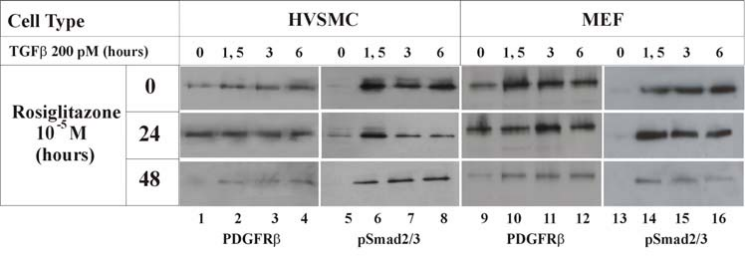
Rosiglitazone treatment decreases expression of PDGFRβ and activation of Smad2/3 (pSmad2/3) *in vitro*. Western blot analysis of activated Smad2/3 and PDGFRβ in whole cell lysates from human VSMC and MEF LRP^+/+^ cells pretreated 0, 24 or 48 hours with rosiglitazone at 10^−5^M and then stimulated with human TGFβ1 (200 pM) for 0, 1.5, 3 or 6 hours.

PPARγ agonists of the thiazolidinedione class have been reported to inhibit TGFβ signaling, CTGF expression and fibrosis [29], [30], and they also can reduce atherosclerosis [31]. We therefore decided to test, whether activation of PPARγ by rosiglitazone, a compound of this class that is in clinical use, might result in reduced nuclear accumulation of pSmad2 and suppression of atherosclerosis in mice lacking LRP1. We first examined the effect of rosiglitazone on PDGFRβ and pSmad2/3 levels in cultured cells in vitro. While TGFβ1 only moderately elevated PDGFRβ expression in vitro, it markedly increased pSmad2/3 levels in cultured human VSMC and in murine fibroblasts. This increase was significantly blunted when the cells were pretreated with rosiglitazone (Figure 2).

**Figure 3:**
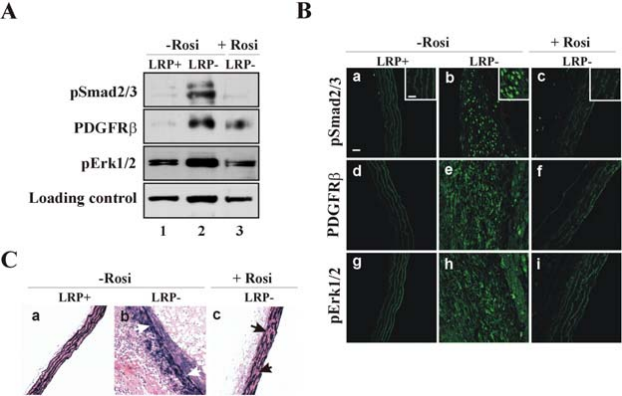
Activation of TGFβ and PDGF signaling in LRP^−^ mouse aortas are both prevented upon rosiglitazone treatment. Mice had been cholesterol-fed for 5 weeks in the absence (−Rosi) or presence (+Rosi) of rosiglitazone (GlaxoSmithKline, 25 mg/kg/day) before analysis. Mouse aortas expressing (LRP^+^) or not expressing (LRP^−^) LRP in VSMC were analyzed by western blot (Panel A) and immunohistochemistry (Panel B) for expression of PDGFRβ (d–f), and for activation of Smad2/3 (pSmad2/3, a–c), and Erk1/2 (pErk1/2, g–i). Panel C shows elastic staining of corresponding sections and gaps in elastic fiber continuity (arrows). Bar indicates 40 µm, insert scale bar in B,a indicates 10 µm.

To test, whether rosiglitazone was able to correct the abnormal accumulation of pSmad2/3 levels and activation of PDGFRβ signaling in smLRP deficient animals in vivo, we fed smLRP^+^ and smLRP^−^ animals a cholesterol-rich diet that either contained or did not contain rosiglitazone for 5 weeks (Figure 3). Aortic extracts were prepared as described earlier [1] and analyzed by immunoblotting (Panel A). Rosiglitazone treatment (lane 3) significantly reduced the elevated pSmad2/3, PDGFRβ and pErk1/2 in smLRP^−^ aortas (lane 2) compared to smLRP^+^ controls (lane 1). We verified this result by immunofluorescence staining of aortas (Panel B). Rosiglitazone treatment (Panels c, f, i) abolished the increased nuclear pSmad2/3, overexpression of PDGFRβ, and the increased pErk1/2 levels in the smLRP^−^ animals (Panels b, e, h). As had been shown previously using the tyrosine kinase inhibitor Gleevec [1], rosiglitazone treatment also considerably improved the integrity of the elastic layers (Panel C).

### Rosiglitazone prevents atherosclerosis in smLRP^−^ mice

**Figure 4:**
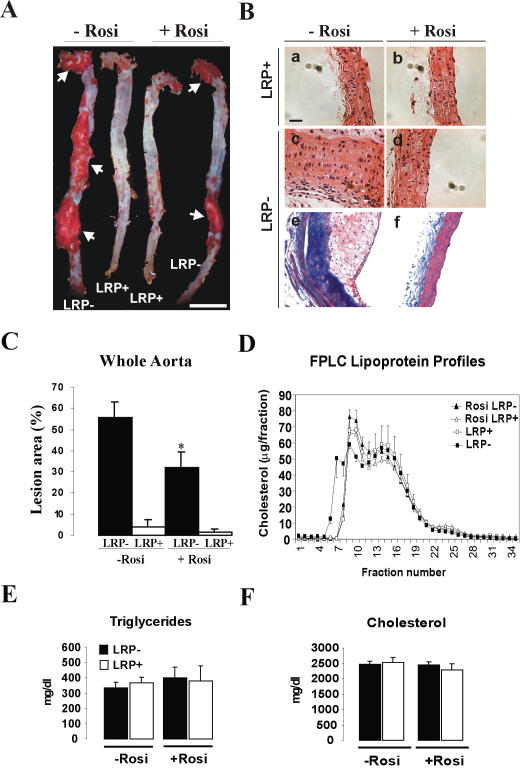
Quantitative analysis of atherosclerotic lesion size in aortas from cholesterol-fed mice with or without rosiglitazone treatment. (A) Aortas from 20-week-old mice that express (LRP^+^) of lack (LRP^−^) LRP in VSMC (n = 6 mice per group). Mice had been cholesterol-fed for 5 weeks in the absence (−Rosi) or presence (+Rosi) of rosiglitazone (GlaxoSmithKline, 25 mg/kg/day) before analysis. Aortae were stained en face with Sudan IV and arrows indicate lipid-laden (Sudan-positive) atherosclerotic lesions. Scale bar, 1.2 cm. (B) Histological analysis of thoracic aortas from animals cholesterol-fed in the absence or presence of rosiglitazone. Hematoxylin and eosin (a and b, LRP^+^; c and d, LRP^−^), and trichrome staining (e and f, LRP^−^) of longitudinal sections. Scale bar in a, 15 µm. (C) Quantitative analysis of atherosclerotic lesion size in aortas from cholesterol-fed LRP^−^ and control (LRP^+^) mice (n = 5 mice per group) with and without rosiglitazone treatment. Values are expressed as mean±s.e.m. *, p<0.05 for LRP^−^ treated versus untreated. (D) FPLC profile of plasma lipoproteins from untreated LRP^−^ (filled squares) and LRP^+^ (opened squares) and rosiglitazone treated LRP^−^ (filled triangles) and LRP^+^ (opened triangles). (E) Plasma triglycerides and (F) cholesterol from untreated and rosiglitazone treated LRP^−^ and LRP^+^ mice. Values are expressed as mean±S.E.M. (n = 10 mice per group).

Thiazolidinediones have been reported to protect male LDLR-deficient mice from developing atherosclerotic lesions [31], coinciding with reductions in gelatinase and TNFα expression. To test whether blockade of the increased Smad activation by rosiglitazone would also reduce the marked atherosclerosis in the smLRP^−^ mice, we quantitatively examined the aortas from the atherogenic diet-fed mice. Atherosclerotic lesion areas were significantly reduced in the presence of the PPARγ agonist (Figure 4A and C), and the striking thickening and fibrosis of the LRP deficient vessel wall was almost completely abolished (Figure 4B). Importantly, these changes were not caused by or secondary to any effect of rosiglitazone on the plasma lipoprotein profile, triglyceride or cholesterol levels (Figure 4D–F).

## Discussion

The findings we have presented here reveal a novel mechanism by which the multifunctional receptor LRP1 functions as an integrator of two distinct cellular signaling pathways in the vascular wall. They also suggest a functional relationship between the molecular events that underlie MFS, the formation of aneurysms and atherosclerosis. Our results show that LRP1 controls Smad2/3-as well as PDGF-dependent signaling in VSMC in a coordinated fashion and by distinct mechanisms.

LRP1 is a target of PDGFRβ-dependent Src kinase activation [2], [32] and we showed earlier that loss of LRP1 expression in VSMCs results in increased PDGFRβ expression and activation [1], [3]. In the present study we have demonstrated, that loss of LRP1 expression also results in increased Smad2/3-dependent signaling in VSMCs. A potent inducer of Smad2 phosphorylation and nuclear translocation is TGFβ [33]. Moreover, induction of PDGF as well as PDGFRβ expression in response to TGFβ stimulation has been recognized as an important component of epithelial-mesenchymal transition, a process that underlies metastasis of some tumors [20] and increases the aggressiveness of others [21]. Our results show that increased pSmad2/3 signaling in the LRP1-deficient vascular wall results in increased PDGFRβ expression and activation, making it likely that TGFβ is directly involved.

Furthermore, LRP1/TGFβR(V), can directly bind TGFβ1 [13], as well as enter into a complex with TGFβR(I) [34]. By binding and endocytosis of the active growth factor and also by sequestering TGFβR(I) away from TGFβR(II), LRP1 is in a central position where it can suppress the normal mode of TGFβ signaling and Smad2/3 activation through the TGFβR(I)/R(II) complex. In addition, LRP1 mediates the endocytosis of TSP1 as well as several matrix metalloproteinases (MMP) from the extracellular space [35]–[37], and TSP1 as well as active MMP2 and MMP9 levels are increased in smLRP deficient aortas (Figure 1, and [1]). TSP1 and MMPs potently and independently activate TGFβ1 [24], [25], [38]. Loss of smLRP would thus be predicted to activate TGFβ/Smad signaling in VSCM by multiple independent mechanisms, and this is consistent with the dramatically increased nuclear pSmad2/3 levels in smLRP^−^ aortas (Figure 1). The resulting disruption of elastic layers, vascular fibrosis, elongation and distension of the aorta, and high incidence of aortic aneurysms directly support the pivotal role of TGFβ signaling in the pathogenesis of MFS and reveal LRP1 as a critical regulator of this pathway in vivo. As such, LRP1 could conceivably contribute as a genetic modifier to the variable severity of expression of MFS in humans.

We have further demonstrated the importance of TGFβ signaling through pharmacological intervention and reversal of the smLRP^−^ phenotype with the PPARγ agonist rosiglitazone. Activation of PPARγ with thiazolidinediones potently inhibits TGFβ signaling in vitro and in vivo [29], [39], [40]. The striking effect of rosiglitazone on the nuclear accumulation of pSmad2/3 and the near complete reversal of the aortic wall thickening and fibrosis, and prevention of atherosclerotic lesion progression confirm the central importance of TGFβ pathway activation for this phenotype. PPARγ agonists might thus complement and enhance the potential therapeutic use of the angiotensin II type 1 receptor blocker Losartan in the treatment of MFS [27].

**Figure 5:**
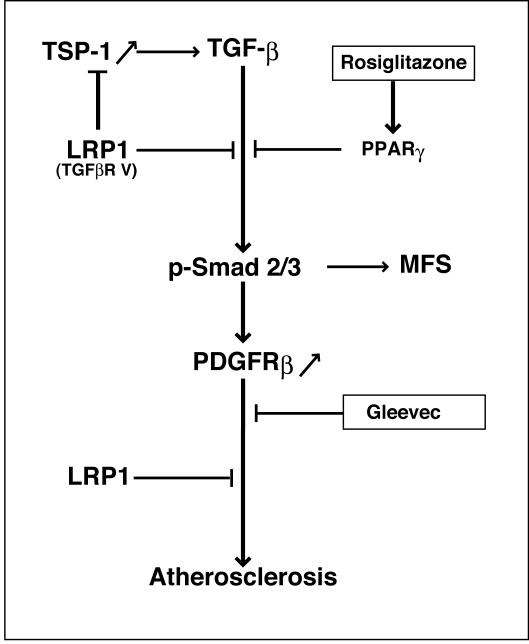
Control of TGFβ and PDGF signaling and protection of vascular wall integrity by LRP1. Absence of LRP1 results in increased activation of TGFβ signaling. This is accompanied by disruption of elastic layers, tortuous aortas and increased fibrosis similar to what is observed in Marfan and Marfan-like syndromes in which the genes for fibrillin-1 or TGFβ receptors are defective. Loss of LRP1 expression leads to increased expression of PDGF receptors. LRP1 also controls PDGFRβ signaling and trafficking through an independent mechanism, and absence of LRP1 promotes VSMC proliferation and severe atherosclerosis. Rosiglitazone blocks TGFβ signaling upstream of PDGFR, which is inhibited by Gleevec. Both drugs are thus effective in reducing arterial wall thickening and atherosclerosis, which are induced by increased PDGFR signaling.

Increased susceptibility to atherosclerosis is not a part of the MFS complex, but a striking feature of smLRP^−^ mice [1]. Likewise, PPARγ agonists are only partially effective against diet-induced atherosclerosis in LDLR deficient mice when LRP1 is functional [31]. By contrast, we have shown that rosiglitazone (this study) as well as the PDGFR tyrosine kinase inhibitor Gleevec [1] are highly potent in protecting smLRP^−^ mice from atherosclerosis. Our findings thus suggest that abnormal activation of PDGFRβ signaling, which has so far not been reported in MFS, is the major mediator of the increased atherosclerosis in the smLRP^−^ mice, and that increased PDGFRβ activity is brought about by two independent and synergistic mechanisms. First, activation of Smad/TGFβ signals in the absence of LRP1 results in the disruption of elastic layers and a tortuous aorta [1], as well as increased expression of PDGF receptors (Figure 2 and 3) [15], [20], [41]. However, since LRP1 also controls PDGFRβ activity and recycling at the level of the plasma membrane [3], loss of LRP1 in smooth muscle cells also results in a greatly increased mitogenic response, VSMC proliferation and the promotion of atherosclerotic lesion formation. This second event is prevented in MFS by the presence of LRP1, which suppresses the abnormal PDGFRβ activation through a posttranslational mechanism [3]. Thus, rosiglitazone (as a Smad/TGFβ antagonist) and Gleevec (as a PDGFRβ inhibitor) act at different steps in a linear sequence of events (Figure 5) that result in a Marfan-like syndrome and culminate in extensive atherosclerosis in smLRP^−^ artery walls.

Nevertheless, blockade of PDGFRβ signaling alone by administration of Gleevec was sufficient to reduce atherosclerotic lesion progression, as well as vascular wall thickness, in smLRP^−^ mice [1], suggesting that signaling through this pathway is necessary for elastic layer disruption and wall thickening. This raises the possibility that PDGFRβ signaling may be activated to some extent in the Marfan vessel wall, or alternatively the mere suppression of its baseline activity may be sufficient to normalize the vessel wall thickening that is initiated by increased TGFβ activity. Consistent with an important role of PDGFRβ in vascular thickening is a recent report that showed transphosphorylation of a truncated PDGFRβ induced by the Angiotensin II type 1 receptor (AT1) [42], which can also activate Smad2-dependent signals independent of TGFβ [28]. AT1 is inhibited by Losartan, a drug that also reduced wall thickening in a mouse model of Marfan syndrome [27]. Taken together, these findings indicate a critical role for Smad2-and AT1-dependent signaling mechanisms in Marfan and Marfan-like syndromes and identify PDGFRβ as another important component of the pathogenic mechanism. Our findings also suggest that rosiglitazone, a clinically approved antidiabetic drug, may be useful for the treatment of vasculopathies that are caused by increased TGFβ signaling, e.g. Marfan syndrome, as well as for the supportive treatment of certain malignancies, such as malignant glioblastomas [43].

## Materials and Methods

### Transgenic mice and diets

Homozygous LDL receptor (LDLR^−/−^) animals LRP deficient in vascular smooth muscle cells (SM22-Cre^+^;LRP^flox/flox^;LDLR^−/−^, referred to as smLRP^−^ or LRP^−^) have been described previously [1] and were maintained on Teklad 6% (w/w) Mouse/Rat Diet 7002 from Harlan Teklad Premier Laboratory Diets (Madison, WI). Six weeks prior to sacrifice, 3–6 months old LRP^−^ and littermate controls (LRP^flox/flox^;LDLR^−/−^, noted LRP^+^) were placed on a high-fat/high-cholesterol (Paigen) diet containing (21% (w/w) milk fat, 1.25% (w/w) cholesterol, 0.5% (w/w) cholic acid) and where indicated, the PPARγ agonist rosiglitazone (Advantia®, GlaxoSmithKline, 25mg/kg/day) was mixed into the diet. All animals used in the experiments were age and sex matched as closely as possible. All animal experiments were conducted according to procedures approved by the institutional Animal Care and Use Committee (IACUC) at UT Southwestern.

### Whole Aorta staining

After 4% paraformaldehyde cardiac perfusion, the whole aortas of animals from the different treated groups were carefully dissected from the heart to the iliac bifurcation. The vessels were rinsed in PBS and dissected to remove connective tissue and attached fat. The vessels were opened longitudinally and stained for lipids with Sudan IV. Atherosclerosis in the aortas was measured by quantitation of Sudan IV–positive lesions as described [44]. Whole mount preparations of the vessels were photographed and digitized. Sudan IV–positive lesions were delineated and analyzed using NIH Image software (NIH, Bethesda, Maryland, USA).

### Histology

Aortic tissue was fixed over night with 4% paraformaldehyde and embedded in paraffin. Longitudinal and transverse sections (10 µm thick) were stained with hematoxylin-eosin under standard conditions.

### Immunofluorescence microscopy

Anesthetized wt and LRP KO mice were perfused through the left cardiac ventricle with warmed Hank's balanced salt solution (20 mM Hepes, pH 7.3) followed by the same solution containing 4% (w/v) paraformaldehyde. Aortas were removed and divided into small pieces. The tissue was immersion-fixed for an additional hour followed by treatment with a mixture of 60% (v/v) methanol, 10% (v/v) glacial acetic acid, 30% (v/v) inhibisol (1,1,1-trichloroethane) for 24 hours. Tissues were then embedded in paraffin. Paraffin sections (5 µm thick) were dewaxed in three changes of xylene (10 minutes each) and rehydrated in PBS. Sections were washed with 50 mM NH_4_Cl in PBS for 30 minutes and blocked by incubation for 1 h in TBS (10 mM Tris-HCl, pH 9.0, 150 mM NaCl) containing 10% (v/v) normal goat serum and 1% (w/v) bovine serum albumin. Samples were then incubated overnight at 4°C with rabbit antiserum raised against pSmad2 (1∶200 dilution), PDGFRβ (1∶100 dilution), phospho-Erk (1∶100 dilution), or preimmune rabbit serum (1∶100 dilution). Sections were washed three times in TBS containing 0.1% bovine serum albumin, and bound primary antibody was detected by incubation for 2 h with Alexa-Fluor 488-conjugated goat anti-rabbit IgG (10 µg/ml, Molecular Probes, Eugene, OR). The tissues slides were washed three times in TBS containing 0.1% bovine serum albumin, rinsed with water, and mounted using Fluorescence Mounting Medium (DakoCytomation). Images were taken using a Leica TCS SP confocal microscope.

### Lipid and Lipoprotein analyses

At sacrifice, blood was collected by cardiac puncture and plasma was analyzed by FPLC on a Superose 6 column (Sigma Chemical Co.). Plasma and liver cholesterol content was determined spectrofluorimetrically as described previously [45].

### Western blotting

MEF LRP deficient cells were seeded in 100 mm dishes (300 000 cells/dish) and grown to confluency in 10 ml DMEM supplemented with 10% (v/v) fetal calf serum and antibiotics. MEF LRP deficient cells were either left untreated or treated for the indicated times with the PPARγ agonist rosiglitazone under reduced serum conditions (0.2% fetal calf serum), followed by stimulation with recombinant human TGFβ1 (200 pM) for 0, 1.5, 3 or 6 hours. Whole cell lysates were then prepared as described previously [1], subjected to SDS polyacrylamide gel electrophoresis, transferred to poly(vinylidene) fluoride (PVDF) membranes (Millipore), and blotted for phospho-Smad2/3 or PDGF receptor-β using specific antibodies directed against PDGF receptor-β and phospho-Smad2/3 (S465/467), respectively (all from Upstate Biotechnology Incorporated, Lake Placid, NY). Proteins were detected using enhanced chemiluminescence (ECL; Amersham Pharmacia Biotech Inc, Piscataway, NJ).
